# Protective Effect of Qiliqiangxin Capsule on Energy Metabolism and Myocardial Mitochondria in Pressure Overload Heart Failure Rats

**DOI:** 10.1155/2013/378298

**Published:** 2013-09-02

**Authors:** Junfang Zhang, Cong Wei, Hongtao Wang, Siwen Tang, Zhenhua Jia, Lei Wang, Dengfeng Xu, Yiling Wu

**Affiliations:** ^1^Integrative Medicine Department, Hebei Medical University, 361 Zhongshan Road, Shijiazhuang 050017, China; ^2^Hebei Yiling Medical Research Institute, Shijiazhuang 050035, China; ^3^Key Research Centre of State Administration of Traditional Chinese Medicine (Collateral Disease of Cardiovascular), Shijiazhuang 050035, China; ^4^Key Laboratory of Collateral Disease of Hebei Province, Shijiazhuang 050035, China; ^5^Yiling Hospital, Hebei Medical University, Shijiazhuang 050091, China

## Abstract

Qiliqiangxin capsule (QL) was developed under the guidance of TCM theory of collateral disease and had been shown to be effective and safe for the treatment of heart failure. The present study explored the role of and mechanism by which the herbal compounds QL act on energy metabolism, *in vivo*, in pressure overload heart failure. SD rats received ascending aorta constriction (TAC) to establish a model of myocardial hypertrophy. The animals were treated orally for a period of six weeks. QL significantly inhibited cardiac hypertrophy due to ascending aortic constriction and improved hemodynamics. This effect was linked to the expression levels of the signaling factors in connection with upregulated energy and the regulation of glucose and lipid substrate metabolism and with a decrease in metabolic intermediate products and the protection of mitochondrial function. It is concluded that QL may regulate the glycolipid substrate metabolism by activating AMPK/PGC-1**α** axis and reduce the accumulation of free fatty acids and lactic acid, to protect cardiac myocytes and mitochondrial function.

## 1. Introduction

Hypertension is one of the most common cardiovascular diseases and a major cause of heart failure. Increase of pressure load due to long-term hypertension will cause myocardial fibrosis and left ventricular compensatory hypertrophy. The accompaning neurohumoral factors, endocrine and metabolic abnormalities further promote ventricular remodeling; structural changes would result in the decline in coronary flow reserve, which eventually leads to heart diastolic and systolic dysfunction [1, 2]. At present, the overall prognosis of heart failure remains less optimistic; thus, further research on the pathogenesis of heart failure and finding new therapeutic targets and measures has become an urgent need in heart failure research. Qiliqiangxin capsule (QL) was developed under the guidance of TCM theory of collateral disease [3]. It is a drug of Chinese medicine for the treatment of heart failure, registered in the Chinese State Food and Drug Administration (SFDA) in 1996, and had been used in clinical practice for more than a decade. Previous studies have shown that QL was able to inhibit Ang II and ALD levels, improve hemodynamics and cardiac function, inhibit ventricular remodeling [4], and reduce the concentration of plasma vasopressin (AVP) and cardiac stress [5]. QL is composed of a number of herbal plants. Previous studies have shown that some of the plants in the QL formula impacted energy metabolism. For example, Astragalus has been shown to inhibit myocardial hypertrophy and improve energy metabolism by increasing the membrane potential [6]. Previous studies have also shown that QL interfered with the energy metabolism related proteins, particularly in increasing the expression of acetyl coenzyme A dehydrogenase and acetal enzyme, indicating that the improvement of heart failure by QL may be related to energy metabolism [3, 7]. However, the relationship between the improvement, by QL, of cardiac dysfunction caused by the chronic pressure overload and energy metabolism and mitochondrial protection remains to be further studied. The present study focused on the cardiac energy metabolism and mitochondrial function and conducted experiments in order to determine the intervention effect of QL on the energy of pressure overload heart failure rats and to further explore its possible mechanism of action by QL.

## 2. Materials and Methods

### 2.1. Drugs and Reagents

**Figure 1:**
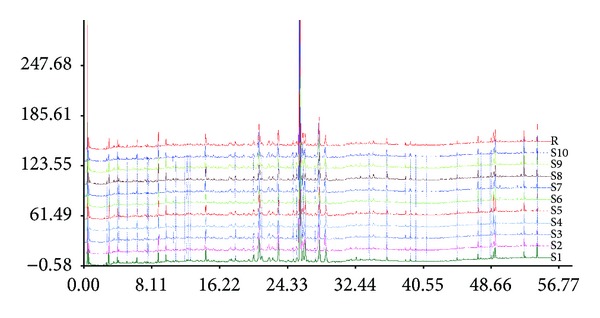
UPLC fingerprints of ten batches of QL capsule.

Qiliqiangxin capsule powder was provided by Shijiazhuang Yiling Pharmaceutical Co. Ltd. The main active pharmaceutical ingredients of QL included Astragalus, Ginseng, *Salvia miltiorrhiza*, Pepperweed Seed, Rhizoma Alismatis, *Polygonatum odoratum*, Ramulus Cinnamomi, *Carthamus tinctorius*, Cortex Periplocae, Tangerine Peel, and other herbs, see Supplementary Material available online at http://dx.doi.org/10.1155/2013/378298. The origin, harvest time, medicinal composites, and processing technology of the herbs were strictly normalized and standardized for different batches of QL capsules. The labeled compounds were verified and standardized according to the Chinese Pharmacopoeia (2005) to achieve quality control of the QL capsule. The aqueous extract of 10 different batches QL was randomly sampled and analysed for the chemical fingerprints by using ultrahigh performance liquid chromatography (UPLC). The similarity analysis of the fingerprints was performed; the similarities were found to be within the range of 0.978 to 1.000, showing that the overall quality of QL had good reproducibility (Figure 1).

Captopril (batch number 11061511) was manufactured by Changzhou Pharmaceutical Factory. Lactic acid detection kit (Jiancheng, Nanjing; batch number 20120710); free fatty acid detection kit (Landau, UK; lot number 234892); adenosine triphosphate ATP (Lot no. 100111202), adenosine diphosphate ADP (Lot no. 10090203190), and rhodamine 123 (Rhodamine-123) were purchased from Sigma Chemical Co., adenosine monophosphate AMP (China National Institutes for Food and Drug Control, batch number 140719-200501), mitochondrial extraction kit (Beijing Solarbio Science & Technology Co., batch number 20120528). Trizol Reagent (Invitrogen); reverse transcriptase (M-MLV), ribonuclease inhibitor (RNasin), dNTP, Taq DNA polymerase, and random primers were purchased from Promega Corporation, USA; Hot Start Fluorescent PCR Core of Reagent Kits (SYBR Green I) was obtained from BBI.

### 2.2. Animal Model and Administration

Male Sprague-Dawley rats (body weight 250–270 g), 150, were provided by the Beijing Vital River Laboratory Animal Technology Co., Ltd (Animal license number: SCXK (Beijing) 2012-0001). The animals were housed at five/cage, fed with standard diet and water ad libitum, and were subject to a 12 h light and 12 h dark cycle. All animal experimental protocols were approved by Animal Care and Use Committee of Hebei Medical University and complied with laboratory animal management and use regulations. Chronic heart failure model was established by thoracic aortic coarctation (TAC) [8], including 135 rats. Rats were intraperitoneal anaesthetized with chloral hydrate (0.35 g/kg) before supine position fixed, intubated, and artificially ventilated. After thoracotomy, descending thoracic aortae were isolated, cleared of fat and connective tissue. Thoracic aortic and polyethylene plastic pipe were tied together securely using a silk string, and then pull out the pipe quickly. The degree of constricting was about 50%. The thorax incision was closed and then stitched with disinfection. The Sham group was with 15 rats which underwent a procedure with thoracic aorta isolation but without coarctation. Four weeks after operation, 75 rats were chosen from the 83 survived ones according to transthoracic echocardiography and ECG and randomly assigned to the following groups: Captopril group (Captopril), orally administered captopril 6.25 mg/kg/d; QL high-dose group (QL-H, *n* = 15), middle-dose group (QL-M, *n* = 15), low-dose group (QL-L, *n* = 15), and the doses of administered QL powder were 1.0, 0.5, and 0.25 g/kg/d, respectively; the volume was 10 mL/kg/d; Model group (TAC, *n* = 15); Sham group (Sham, *n* = 15); Sham group and the test group were administered with an equal volume of 0.5% sodium carboxymethyl cellulose (CMC-Na), orally administered for 6 weeks.

### 2.3. Heart Weight Index and Hemodynamic Measurement

After treatment for six weeks, the animals were anesthetized to perform the separation of the right carotid artery and cannulation. Left ventricular systolic pressure (LVSP) and left ventricular pressure (LVP) were measured by using BL-420E polygraph (Chengdu TaiMeng Technology Corp., LTD). After collecting blood from the abdominal aorta, the heart was rapidly removed, rinsed with cold physiological saline, and water adsorbed by filter paper. The excess tissues around the hearts were removed, and hearts were weighed on a balance and then immediately put into liquid nitrogen for storage. Heart weight index was calculated using the formula: index = heart weight (HW)/body weight (BW).

### 2.4. Content of Serum Lactic Acid (LA) and Free Fatty Acid (FFA)

Blood was stored at room temperature for 2 h and centrifuged for 10 min at 3500 rpm; the serum was then loaded into EP tube. The optical density value was measured by using the XD711 ELISA analyzer (Shanghai Xun-Da Medical Instrument Corporation Ltd.) according to the experimental method specified in the instruction of lactic acid detection kit; serum free fatty acids were measured by using Hitachi 7080 biochemical analyzer.

### 2.5. Mitochondrial Transmembrane Potential (MMP)

About 0.3 g of fresh myocardial tissue was prepared and rinsed by physiological saline. Myocardial tissue was sheared and washed with PBS buffer. Cell mass was filtered with 300 mesh metal mesh and single cell suspension was collected. The supernatant was discarded after centrifugation (1000 rpm, 5 min). Single cell suspension was adjusted to 1 × 10^5^/mL in the culture medium. Rhodamine 123 was used as fluorescent probe (final concentration of 0.5 *μ*mol/L). The suspension was incubated for 15 min in the dark place, and then the changes in the myocardium mitochondria MMP were measured using flow cytometer (EPICS-XL II, Beckman Coulter, USA) with excitation wavelength and emission wavelength at 480 nm and 530 nm, respectively.

### 2.6. Mitochondria Extract and Determination of Oxidative Respiratory Function

Fresh myocardial tissue was collected at the end of the experiment and extracted with mitochondrial extraction kit to prepare mitochondrial suspension by differential centrifugation at low temperature. Protein content in myocardial mitochondrial suspension was determined by Coomassie brilliant blue. 2.5 mL of GENMED medium liquid was added into the reaction glass tank, mixed, and sealed, and the mitochondria, State IV substrate solution and State III substrate solution were added in proper sequence as instructed by the manufacturer. Mitochondrial State III and State IV respiration rate in the closed reaction system were determined using dissolved oxygen electrolytic analyzer (ORION 4 STAR, the U.S. Thermo Electron), and mitochondrial respiratory control ratio (RCR) was calculated according to the following formula: RCR = State III respiration rate/State IV respiration rate.

### 2.7. Adenine Nucleotide ATP, ADP and AMP Levels and Energy Charge in Myocardial Tissue

The cryopreserved myocardial tissues were weighed and added 0.4 mol/L precooling perchloric acid at 5 mL/g ratio, homogenized on the ice bath, and then centrifuged at 4000 r/min for 10 min at 4°C. Supernatant (400 *μ*L) was collected and added 25 *μ*L 2 mol/L to adjust pH to 6.5, centrifuged again at 4000 r/min for 10 min at 4°C, and the supernatant was filtered with a 0.45 micron membrane. The supernatant preparation was loaded onto a 10 A high performance liquid chromatography detector (Shimadzu Corporation, Japan), followed by separation and detection at wavelength of 254 nm. ATP, ADP, and AMP levels were calculated according to the elution peak area and standard concentrations. The results were expressed at *μ*mol/g tissue. Total adenylate pool (ATP + ADP + AMP) and the energy charge [EC = (ATP + 0.5 × ADP)/(ATP + ADP + AMP)] were calculated.

### 2.8. Real-Time RT-PCR

Total RNA of myocardial tissue was extracted using Trizol, which was then reverse transcribed into double stranded cDNA. Real-time RT-PCR was carried out using a thermocycler (ABI 7300 Real-Time PCR System, USA). PCR thermal cycling parameters were as follows: the denaturing step at 96°C for 4 min, followed by 40 cycles annealing step at 94°C 30 s, 58°C 30 s, and 72°C 30 s. Fluorescence signal was collected in each cycle of the third step 72°C 30 s. Using GAPDH gene as an internal reference and by comparing target gene expression and with Control group, the relative quantitative value (RQ value) was calculated and used for statistical analysis. The primer sequences were shown in Table 1.

### 2.9. Western Blot Analysis

Myocardial tissue about 100 mg and 1ml precooled lysate were homogenised in ice bath. Supernatant was collected after centrifugation (4°C, 8000 rpm and 10 min), and the protein concentration of supernatant, measured by Nanodrop 2000 spectrophotometer, was adjusted to the final concentration of 1%. The samples were mixed with equal volumes of 5 × SDS sample buffer, and then boiled for 5 min, centrifuged, and loaded onto 12% SDS-PAGE for electrophoresis. After being transferred onto a polyvinylidene difluoride membrane (PVDF), the membranes were blocked with 5% milk tris-buffered saline-tween 20 and probed with primary antibody (1 : 1000) and HRP conjugated secondary antibody (1 : 10000), separated by extensive washings. The membrane was treated with enhanced chemiluminescence substrate and the bands on the membrane were visualized and analyzed using UVP BioImaging System.

### 2.10. Statistical Methods

All data were presented as mean ± standard deviation, single factor analysis of variance (ANOVA) was performed with the statistical software SPSS 17.0, Dunnertt'sT3 was used for unequal variances, and the difference was statistically significant at *P* < 0.05.

## 3. Results

### 3.1. Changes in LVP, LVSP and Heart Weight Index

The rats in each operated group manifested low activity, short of breath, poor appetite, listlessness and other symptoms of heart failure. Compared with Sham group, LVP, LVSP and heart weight indices of the rats in Model group were significantly increased (*P* < 0.01). Compared with Model group, the intervention of six weeks' administration with QL reduced the LVP and LVSP levels to some extent. Model group increased significantly compared with Sham group. Each treatment group had significantly lower heart weight index (*P* < 0.05), especially in the QL-H and Captopril group in which the heart weight was significantly lower (*P* < 0.01) (Table 2).

### 3.2. Serum LA and FFA Levels in Each Group

**Figure 2:**
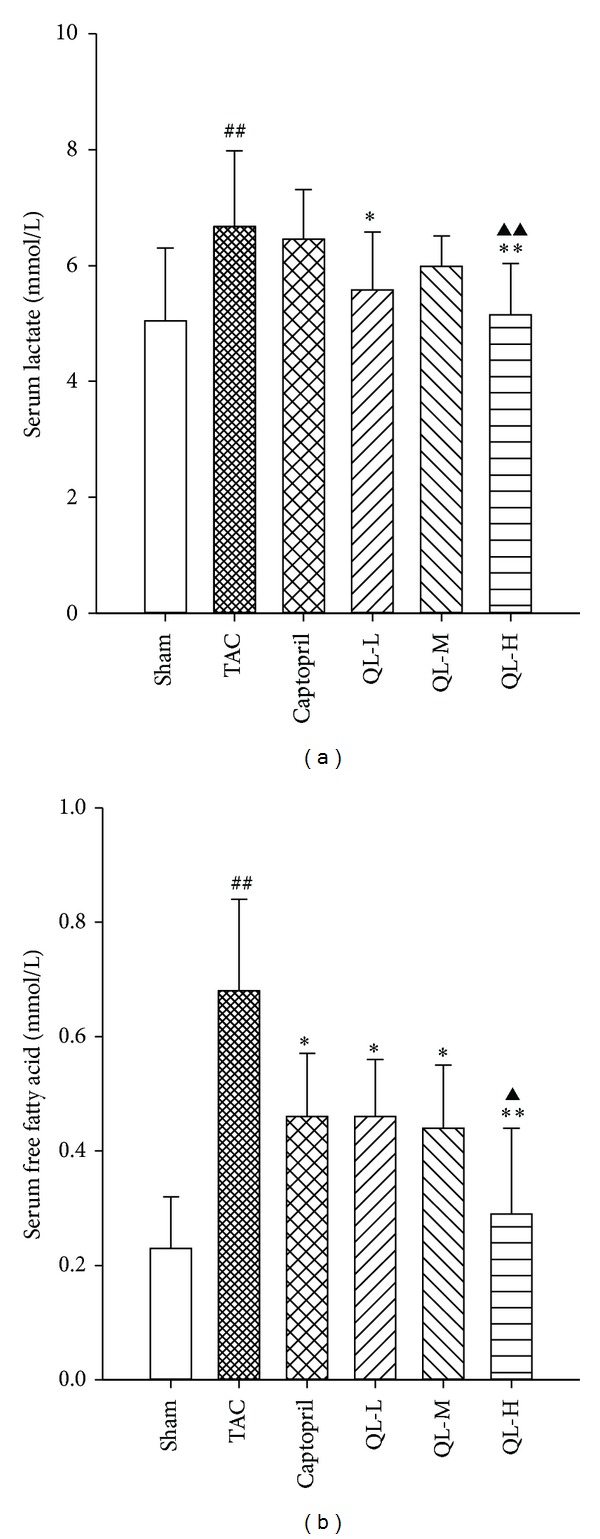
Serum lactate and free fatty acid content in all the six groups. (a) Serum lactate concentrations in the myocardium. (b) The content of serum free fatty acid in the myocardium. Rats were administered with vehicle (0.5% CMC-Na), QL (0.25 g/kg/d, 0.5 g/kg/d, 1 g/kg/d), or Captopril (6.25 mg/kg/d). ^##^ *P* < 0.01, compared TAC group with Sham group; *P < 0.05, **P < 0.01, compared respective dose group with TAC group; ^▲^ *P* < 0.05, ^▲▲^ *P* < 0.01, compared QL respective dose group with Captopril group.

As shown in Figures 2(a) and 2(b), serum lactate and free fatty acids levels in Model group were higher compared with Sham group (*P* < 0.01). Low dose and high dose groups of QL reduced serum lactate level in the pressure overload rat (*P* < 0.05); the high-dose group significantly lowered serum lactate level (*P* < 0.01). Although the positive group also reduced the lactic acid level, the difference was not significant. Compared with Model group, free fatty acid level was significantly lower in Captopril group and each dose group of QL (*P* < 0.05). The high dose group had a highly significant difference (*P* < 0.01).

### 3.3. Changes in Mitochondrial Membrane Potential in Cardiac Myocytes and Respiratory Function

**Figure 3:**
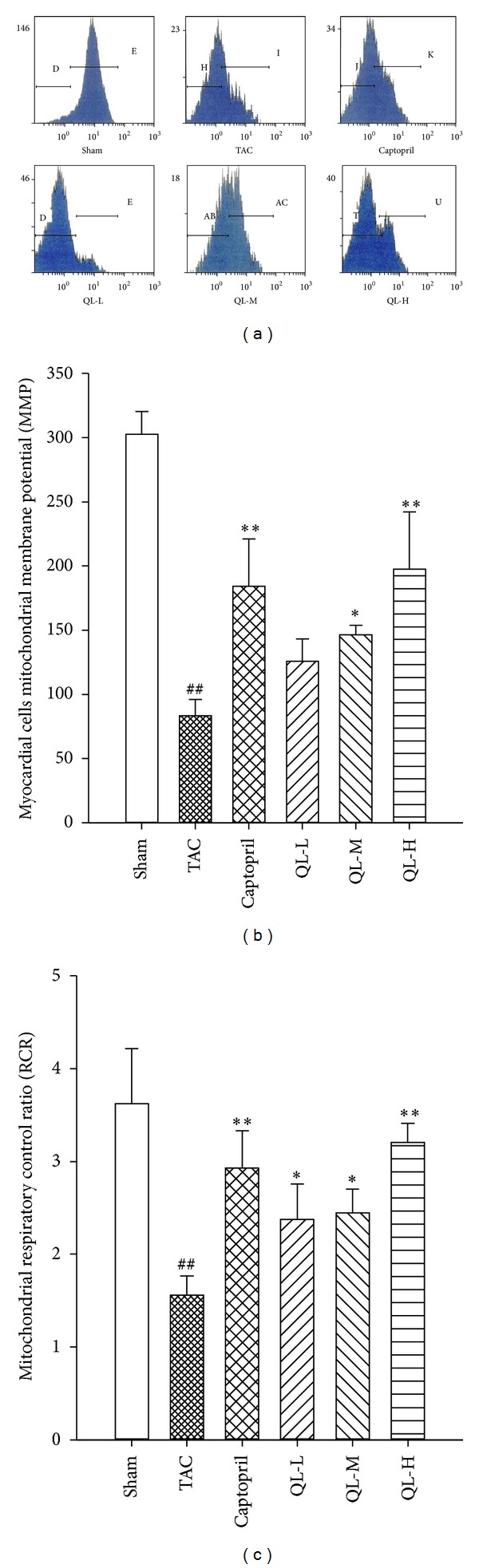
The measurement of myocardial cells mitochondrial membrane potential (MMP) and respiratory control ratio (RCR). (a) The measurement of myocardial cells mitochondrial membrane potential. (b) Comparison of MMP in different groups. (c) Comparison of mitochondria respiratory control ratio (RCR) in different groups. ^##^ *P* < 0.01, compared TAC group with Sham group; *P < 0.05, **P < 0.01, compared respective dose group with TAC group.

Compared with Sham group, myocardial mitochondrial membrane potential was declined after model establishment. After drug intervention for six weeks, myocardial mitochondrial membrane potential was significantly elevated compared with Model group (*P* < 0.05) (Figures 3(a) and 3(b)). Each treatment group showed an improved mitochondrial respiratory function and increased respiratory control ratio (*P* < 0.05). The effects of Captopril group and QL-H group were highly significant (*P* < 0.01), as shown in Figure 3(c).

### 3.4. Total Adenylate Pool and Energy Charge Level in Myocardial Tissue

**Figure 4:**
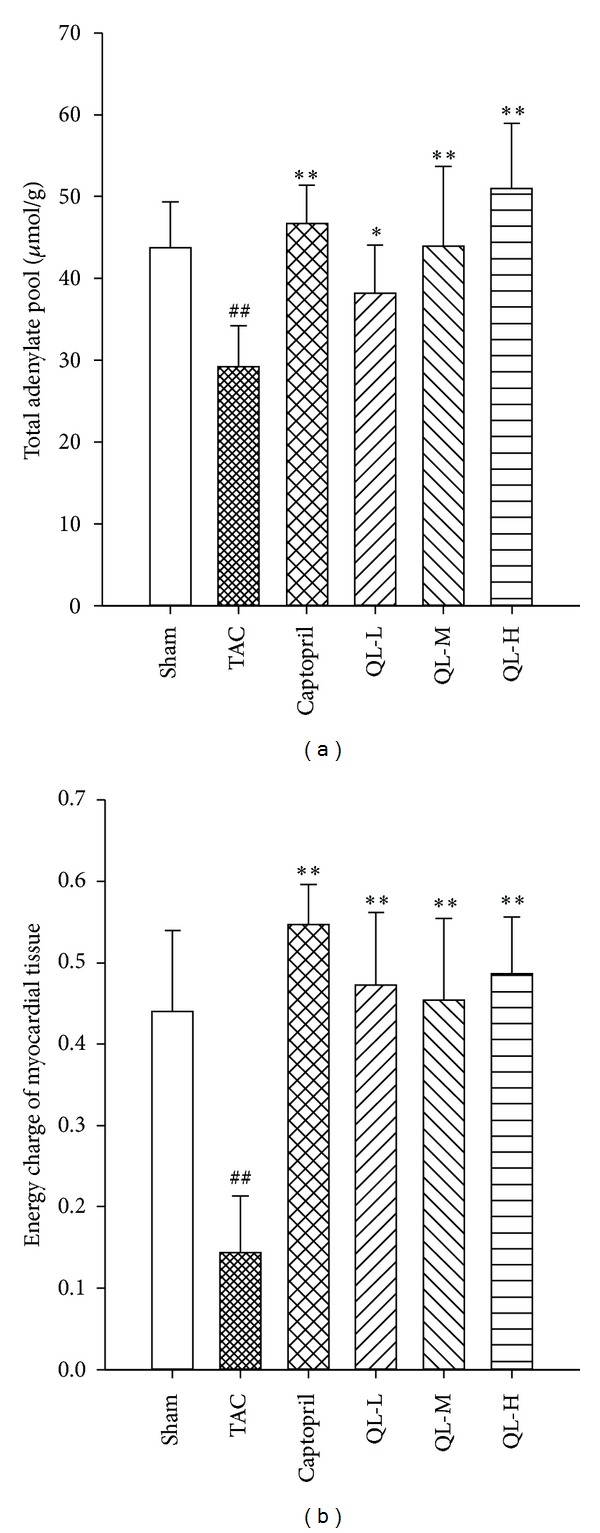
Total adenylate pool and energy charge of myocardial tissue in rats. (a) Effects of QL on the total adenylate pool in myocardial tissue. (b) Effects of QL on energy charge of myocardial tissue in rats. ^##^ *P* < 0.01, versus TAC with Sham; *P < 0.05, **P < 0.01, versus QL respective dose group with TAC.

Compared with Sham group, total adenylate and energy charge values in Model group were significantly lower (*P* < 0.01), showing a disturbance of myocardial energy supply occurred in postoperative rats; the ventricular reconstruction was accompanied by metabolic remodeling. Compared with Model group, the drug intervention group significantly increased the adenosine pool and energy charge value; total adenosine pool and energy charge value were higher in QL-L group than Model group (*P* < 0.05, *P* < 0.01, resp.). The increase of total adenylate and energy charge value in myocardial tissue was most notable in Captopril group and QL-H group (Figure 4).

### 3.5. Real-Time PCR Results in Myocardial Tissue

**Figure 5:**
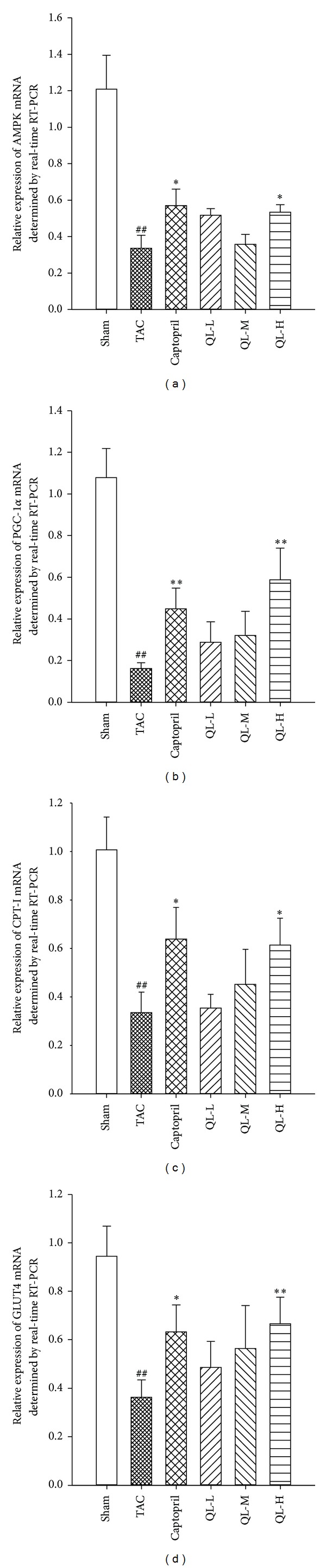
Real-time reverse transcription RT-PCR results. (a) The expression of AMPK mRNA in the cardiac tissue in the six groups was determined by real time RT-PCR. (b) The relative expression of PGC-1*α*mRNA in the myocardium of the six groups was determined by real time RT-PCR. (c) Compared the relative expression of CPT-I mRNA determined by real time RT-PCR. (d) The expression of GLUT4 in the myocardium was determined by real time RT-PCR. ^##^ *P* < 0.01 versus TAC with Sham; *P < 0.05, **P < 0.01, versus respective dose group with TAC.

Compared with Sham group, the expression levels of AMPK, PGC-1*α*, CPT-I, and GLUT4 mRNA were significantly lower in Model group (*P* < 0.01); the relative expression levels of each group indicator were increased to some extent after drug intervention. The expression levels of AMPK, PGC-1*α*, CPT-I, and GLUT4 mRNA were significantly different in QL-H group and Captopril group compared with the Model group (*P* < 0.05, *P* < 0.01, resp.) (Figure 5).

### 3.6. Western Blot Results in Myocardial Tissue

**Figure 6:**
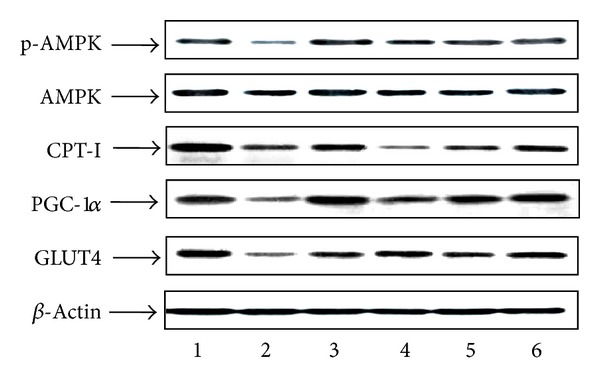
The p-AMPK, AMPK, PGC-1*α*, CPT-I and GLUT4 protein expressions in myocardial tissue of different groups. 1: Sham group; 2: TAC group; 3: Captopril group; 4: QL-L group; 5: QL-M group; 6: QL-H group.

Compared with Sham group, the protein expression of PGC-1*α*, CPT-I, and GLUT4 in Model group decreased significantly (*P* < 0.05, *P* < 0.01, resp.). Compared with Model group, the above protein expression in treatment group increased at varying degrees (*P* < 0.05, *P* < 0.01). Compared with Sham group, the protein expression of p-AMPK and AMPK in Model group showed no statistical difference (*P* > 0.05). Compared with Model group, the expression level of p-AMPK in treatment group increased significantly, in which the QL-M and QL-H group increased obviously (*P* < 0.01). The total amount of AMPK in each group did not show significant difference (*P* > 0.05) (Table 3, Figure 6).

## 4. Discussion

QL capsules are traditional Chinese medicine (TCM) formula, which was developed according to TCM theory. The QL has extracts obtained from 11 herbs. Pharmacological studies have found that QL contains a number of active substances such as ginseng saponin, astragalus saponin, flavonoid, cardenolide, and phenolic acid. There is a significant effect in the clinical treatment of chronic heart failure as shown in previous studies [9, 10].

Failure or hypertrophic myocardium caused imbalance between demand increase and production decrease of ATP, which was the root cause of decrease in myocardial metabolic reserve and cardiac function degradation [11, 12]. The condition is reflected by the decrease of myocardial ATP level in the failing heart, mitochondrial dysfunction, and imbalance of carbohydrate and fatty acid oxidation [13]. 

Myocardial remodeling can decrease oxygen level in the myocardial tissue and affects substrate oxidation, thus resulting in the reducing of aerobic metabolic efficiency and the shift of energy supply to anaerobic glycolysis. In this case, free fatty acids (FFA) and glucose aerobic oxidation are decreased, and insulin resistance may occur [14, 15]. The increase in lactic acid and FFA levels within the cells may result in cardiac toxicity and exacerbation of energy metabolism [16]. Changes in myocardial substrate metabolism in heart failure reduce the ATP level and also cause the increase of lactic acid and the accumulation of free fatty acids, leading to intracellular acidosis and increasing cardiac myocytes injury. This is consistent with the experimental results from the present study. QL lowered fatty acids and free fatty acids levels, which was closely related to substrate metabolism. We further detected the expression levels of carnitine acyltransferase enzyme-I (CPT-I) and glucose transporter protein 4 (GLUT4), which were key factors for fatty acids and glucose oxidation, respectively. Thus QL improved the expression levels of CPT-I and GLUT4. These data illustrated that QL promoted the oxidation of fatty acids and glucose by optimizing and regulating the glycolipid substrate metabolism, and decreasing the accumulation of lactic acid and free fatty acids, thereby protecting the cardiac myocytes.

The energy metabolism in cardiac myocytes is closely related to mitochondrial function status. Mitochondrial damage and significant reduction of oxidative phosphorylation efficiency can be caused by ischemia and hypoxia and the intermediate metabolite accumulation in failing heart. There is an internal negative and external positive transmembrane potential difference in mitochondrial inner-membrane. When the membrane potential decreases uncoupling of oxidative phosphorylation, ATP depletion and increase in oxygen radicals take place, thereby inducing cardiac myocytes into the irreversible process of apoptosis. High-energy phosphate is a direct source of energy for maintaining normal life activities of myocardial cells. The adenylate pool reflected the energy reserve state and the energy metabolic state. The results from the present study showed that, compared with Sham group, mitochondrial adenylate pool and energy charge value, RCR, and membrane potential were significantly lower in Model group, which were the same as the results of Ingwall [11], indicating that mitochondria and substrate oxidation were damaged, and energy reserves and utilization were reduced. However, the administered group improved the above indicators, particularly for the high QL dose group which had improved obviously energy metabolism. Taken together, those data indicate that the effect of QL on protecting mitochondria and improving energy metabolism is one of the important mechanisms of improving cardiac function and delaying heart failure development process. 

AMP-activated protein kinase (AMPK) is a key modulator of lipid and glucose metabolism and energy balance. When ATP level is reduced, AMPK is rapidly activated, and becomes involved in the cell energy regulation [17]. AMPK antagonized lipotoxicity in endothelial cells due to FFA by promoting the oxidation of endothelial cell on FFA [18], while the myocardial glucose uptake and GLUT4 translocation are increased. Activated AMPK not only inhibits fatty acid synthesis, but it also enhances fatty acid oxidation by reducing the levels of malonylCoA, which is a allosteric inhibitor of CPT-I [19]. Furthermore, AMPK stimulates GLUT4 translocation and increasing whole-body utilization of glucose [20]. PGC-1*α* is a key nuclear receptor coactivator that can induce mitochondrial biogenesis, modulate number and mass of mitochondrial, fatty acid oxidation, and thermogenesis [21]. The current studies confirmed that QL activated AMPK and upregulated the expression of PGC-1*α*, compared with the Model group. Thereby, QL regulated energy metabolism by activating AMPK/PGC-1*α* signal factor. Specifically, QL regulated fatty acid and glucose metabolism, reduced the levels of FFA and LA through activation of AMPK. The improved mitochondrial respiratory function by QL has a relationship with its regulation of PGC-1*α* and the PGC-1*α* downstream. PGC-1*α* has many biological effects, for example, through the TR*β*1, NRFS, and ERRS it affects mitochondrial biogenesis [22]. Previous studies have shown that AICAR, AMPK-activating compound, could induce upregulation of PGC-1*α* mRNA [23]. AMPK/PGC-1*α*, a signaling pathway involved in energy metabolism, was therefore the pathway regulated by QL.

In summary, the improvement of cardiac energy metabolism is an important part of delaying heart failure progression. QL reduced heart weight index of rat model of TAC-induced pressure overload, improved hemodynamic parameters, and was related to the increase of myocardial high-energy phosphate content as well as the improvement of energy reserves and the metabolic state. We argue that QL may regulate the glycolipid substrate metabolism by activating AMPK/PGC-1*α* axis and reduce the accumulation of free fatty acids and lactic acid, to protect cardiac myocytes and mitochondrial function.

## Supplementary Material

QL is composed of a number of herbal plants. As the chemical components of this medicine are complex and varied, liquid chromatography mass spectrometry (LC-MS/MS) was adopted to study chemical component, and nearly 200 compounds were identified. Toxicological study showed the security of clinical application.

## Figures and Tables

**Table 1:** Primer for RT-PCR.

Gene	bp	GenBank ID	Primer Sequence (5′ to 3′)
GAPDH	120	NM_017008	S: TGAACGGGAAGCTCACTGG
			A: GCTTCACCACCTTCTTGATGTC

CPT-I	87	NM_013200.1	S: CCA GGC AAA GAG ACA GAC TTG
			A: GCCAAACCTTGAAGAAGCGA

GLUT4	62	NM_012751	S: CCC ACA AGG CAC CCT CAC TA
			A: TGC CAC CCA CAG AGA AGA TG

AMPK	133	NM_019142.1	S: ACA GAA GCC AAA TCA GGG ACT
			A: CAC GGA TGA GGT AAG AGA GAC T

PGC-1*α*	168	NM_031347	S: AGC CAC TAC AGA CAC CGC AC
			A: CCT TTC AGA CTC CCG CTT C

GAPDH: glyceraldehydes 3-phosphate dehydrogenase; CPT-I: carnitine palmitoyl transferase I; GLUT4: Glucose transporter 4; AMPK: AMP-activated protein kinase; PGC-1*α*: peroxisome proliferators-activated receptor-*γ* coactivator-1*α*; MW: molecular weight.

**Table 2:** The changes of body weight, heart weight/body weight ratio (HW/BW), LVP, and LVSP.

Group	Heart weight (mg)	Body weight (g)	HW/BW (mg/g)	LVP (mmHg)	LVSP (mmHg)
Sham	1.32 ± 0.12	499 ± 49.3	2.40 ± 0.11	110 ± 21.0	105 ± 16.7
TAC	1.40 ± 0.14	490 ± 41.2	3.02 ± 0.47^##^	144 ± 23.9^##^	133 ± 23.2^##^
Captopril	1.36 ± 0.10	492 ± 35.2	2.64 ± 0.25*	127 ± 18.6**	116 ± 19.1*
QL-L	1.33 ± 0.14	500 ± 52.4	2.67 ± 0.31*	127 ± 13.9*	117 ± 18.6
QL-M	1.31 ± 0.20	503 ± 46.2	2.61 ± 0.35*	125 ± 18.7**	115 ± 13.9*
QL-H	1.28 ± 0.13	490 ± 42.0	2.54 ± 0.29**	119 ± 13.9**	103 ± 16.0**

Sham: control group; TAC: model group; QL-L: low-dose QL group; QL-M: medium-dose QL group; QL-H: high-dose QL group;

The Sham rats and TAC rats were treaded with vehicle (0.5%CMC-Na, 10 mL/kg/d); the low-dose QL group was treated with 0.25 g/kg/day QL: the medium-dose QL group was treated with 0.5 g/kg/day QL; the high-dose QL group was treated with 1 g/kg/day QL. Captopril group was treated with 6.25 mg/kg/day Captopril. Values were expressed as mean ± standard deviation. Compared with TAC, ^#^ *P* < 0.05, ^##^ *P* < 0.01; compared with TAC, *P < 0.05, **P < 0.01.

**Table 3:** The p-AMPK, AMPK, PGC-1*α*, CPT-I, and GLUT 4 protein expressions in myocardial tissue of different groups.

Group	p-AMPK	AMPK	PGC-1*α*	CPT-I	GLUT 4
Sham	0.50 ± 0.12	0.67 ± 0.15	0.80 ± 0.12	1.15 ± 0.20	0.70 ± 0.14
Model	0.31 ± 0.14	0.45 ± 0.17	0.29 ± 0.07^##^	0.57 ± 0.10^#^	0.39 ± 0.18^#^
Captopril	0.79 ± 0.22**	0.57 ± 0.36	0.76 ± 0.09**	1.03 ± 0.32*	0.71 ± 0.27*
QL-L	0.68 ± 0.24*	0.49 ± 0.16	0.35 ± 0.03	0.65 ± 0.17	0.62 ± 0.02
QL-M	0.79 ± 0.11**	0.44 ± 0.14	0.53 ± 0.06**	0.93 ± 0.27	0.75 ± 0.15*
QL-H	0.94 ± 0.13**	0.63 ± 0.22	0.75 ± 0.03**	1.07 ± 0.36*	0.81 ± 0.14**

Sham: control group; TAC: model group; QL-L: low-dose QL group; QL-M: medium-dose QL group; QL-H: high-dose QL group; values were expressed as mean ± standard deviation. Compared with TAC, ^#^ *P* < 0.05, ^##^ *P* < 0.01; compared with TAC,*P < 0.05, **P < 0.01.
