# Supplementary material for: Protective Effect of Qiliqiangxin Capsule on Energy Metabolism and Myocardial Mitochondria in Pressure Overload Heart Failure Rats
Source: Evid Based Complement Alternat Med. 2013 Sep 2;2013:378298. doi: 10.1155/2013/378298 (PMC3775405; doi:10.1155/2013/378298)
Supplement: Supplementary file 1 — QL is composed of a number of herbal plants. As the chemical components of this medicine are complex and varied, liquid chromatography mass spectrometry (LC-MS/MS) was adopted to study chemical component, and nearly 200 compounds were identified. Toxicological study showed the security of clinical application. [file 378298.f1.pdf]

QL is composed of a number of herbal plants. As the chemical components of this medicine are complex and varied, liquid chromatography mass spectrometry (LC-MS/MS) was adopted to study chemical component, and nearly 200 compounds were identified. Toxicological study showed the security of clinical application.
